# A functional assembly framework based on implementable neurobionic material

**DOI:** 10.1002/ctm2.277

**Published:** 2021-01-13

**Authors:** Xiang Zou, Conglin Jiang, Yirui Sun, Donghua Zhao, Yusheng Tong, Ying Mao, Liang Chen

**Affiliations:** ^1^ Department of Neurosurgery, Huashan Hospital Fudan University Shanghai China; ^2^ Neurosurgical Institute of Fudan University Shanghai China; ^3^ Shanghai Clinical Medical Center of Neurosurgery Shanghai China; ^4^ Shanghai Key Laboratory of Brain Function and Restoration and Neural Regeneration Shanghai China; ^5^ Tianqiao and Chrissy Chen International Institute for Brain Diseases Shanghai China; ^6^ Department of Mathematics Fudan University Shanghai China

**Keywords:** decision making, implementable device, neurobionic material, short‐term memory

## Abstract

Neurobionic material is an emerging field in material and translational science. For material design, much focus has already been transferred from von Neumann architecture to the neuromorphic framework. As it is impractical to reconstruct the real neural tissue solely from materials, it is necessary to develop a feasible neurobionics framework to realize advanced brain function. In this study, we proposed a mathematical neurobionic material model, and attempted to explore advanced function only by simple and feasible structures. Here an equivalent simplified framework was used to describe the dynamics expressed in an equation set, while in vivo study was performed to verify simulation results. In neural tissue, the output of neurobionic material was characterized by spike frequency, and the stability is based on the excitatory/inhibitory proportion. Spike frequency in mathematical neurobionic material model can spontaneously meet the solution of a nonlinear equation set. Assembly can also evolve into a certain distribution under different stimulations, closely related to decision making. Short‐term memory can be formed by coupling neurobionic material assemblies. In vivo experiments further confirmed predictions in our mathematical neurobionic material model. The property of this neural biomimetic material model demonstrates its intrinsic neuromorphic computational ability, which should offer promises for implementable neurobionic device design.

## Graphical abstract

The neurobionic assembly model based on our simplification is a feasible framework to realize, and can achieve some brain functions. Our findings may provide inspiration for implementable neurobionic device design.

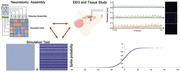

## 1 INTRODUCTION

Neurobionic material is the emerging field in material and translational science. Over the past decades, developing electronics have emerged to mimic biological sensory and motor systems.^1^, ^2^, ^3^, ^4^ Nonetheless, no artificial devices are readily available to mimic advanced biological functions such as decision making or short‐term memories. Further development of those functional artificial devices may rely on progress in material, fabrication, and advanced algorithm.^5^, ^6^, ^7^ Recent development in artificial intelligence and big data analysis drives an ever‐rising desire for superior computing algorithm and associated hardware. However, the current von Neumann architecture computational system limits computing speed caused by data shuttling among hardwires.^8^ Thus, cognitive computers of non‐von Neumann architecture has been developed to overcome this bottleneck, such as neuromorphic computing.^9^, ^10^ Recent progress in phase‐change random‐access memory (PCRAM) device is one candidate for programmable material.^11^, ^12^ In this context, it is necessary to develop a feasible neurobionics framework to conceptualize advanced brain function.

Brain is a natural computational organ adapt to the environment. The network is the most distinctive mesoscopic structure of brain. Although more details for brain structure have been revealed over the past century following findings by Camillo Golgi, the understanding achieved is still not very in‐depth. The wiring logic of brain tissue can be the template of neurobionic material. Generally, function of neurobionic material depends on the framework. However, as the neurobionic material cannot exactly duplicate the real tissue, we should maximize the effect by a feasible artificial framework. Tsien and associates^13^, ^14^, ^15^ raised a postulate regarding the cerebral basic wiring logic, indicating that the brain may organize the microarchitecture of cell assemblies as 2*n*‐1 combinations that would enable knowledge and adaptive behaviors to emerge upon learning. This hypothesis suggests certain neural assembly as a classifier, which is the basis of advanced functions.

In spite of all these particulars, we want to verify the advanced capability of the simple network property, in order to fit the feasible neurobionics framework. In this study, we proposed a neurobionic material model, and attempted to explore possible computational function with only the structure of the network that is achievable by PCRAM. Accomplishment of such neurobionic material model should offer promises for implementable neurobionic device design and translational science field.

**HIGHLIGHTS**

1. We mathematically prove the meaning of synapses as the parameter of a nonlinear equation set, in which spike frequency of each neuron can meet the solutions.
2. Some advanced brain functions are feasible in this neurobionic material model.
3. The model based on our simplification is practical in biomaterial field.

## 2 MATERIALS AND METHODS

### 2.1 Mathematical model from neural tissue to neurobionic material

To fit the feasibility needs from material design, we should simplify the structure of the artificial assembly, but with little discount of its computational ability. The simplest model of a neuron is a summator, which can sum up the input signal, judge, and then output a signal known as a spike. Even in this momentary process, thousands of reactions have happened, but all those details are ignored in this study except the most indispensable property. This kind of network model was first described by McCulloch and Pitts.^16^ It is noteworthy that the neuro‐behavior is discontinuous; the neuron's output is represented by the density or probability of spikes, not the amplitude. Actually, the behavior of the neuro‐network can be divided by a very short time interval, proposed as a spike unit. This is a sound approximate assumption that makes the following study possible. A real neuro‐network is extremely complicated; for example, different connections, synapse weights, and thresholds are all unknowable. However, all those factors can be discretized and made equivalent to other forms. Now, we raise two equivalent and two discretized assumptions, aiming to transform the extremely complicated neuro‐network behavior into a describable mathematical model, which is similar to the Hopfield network model.^17^

**FIGURE 1:**
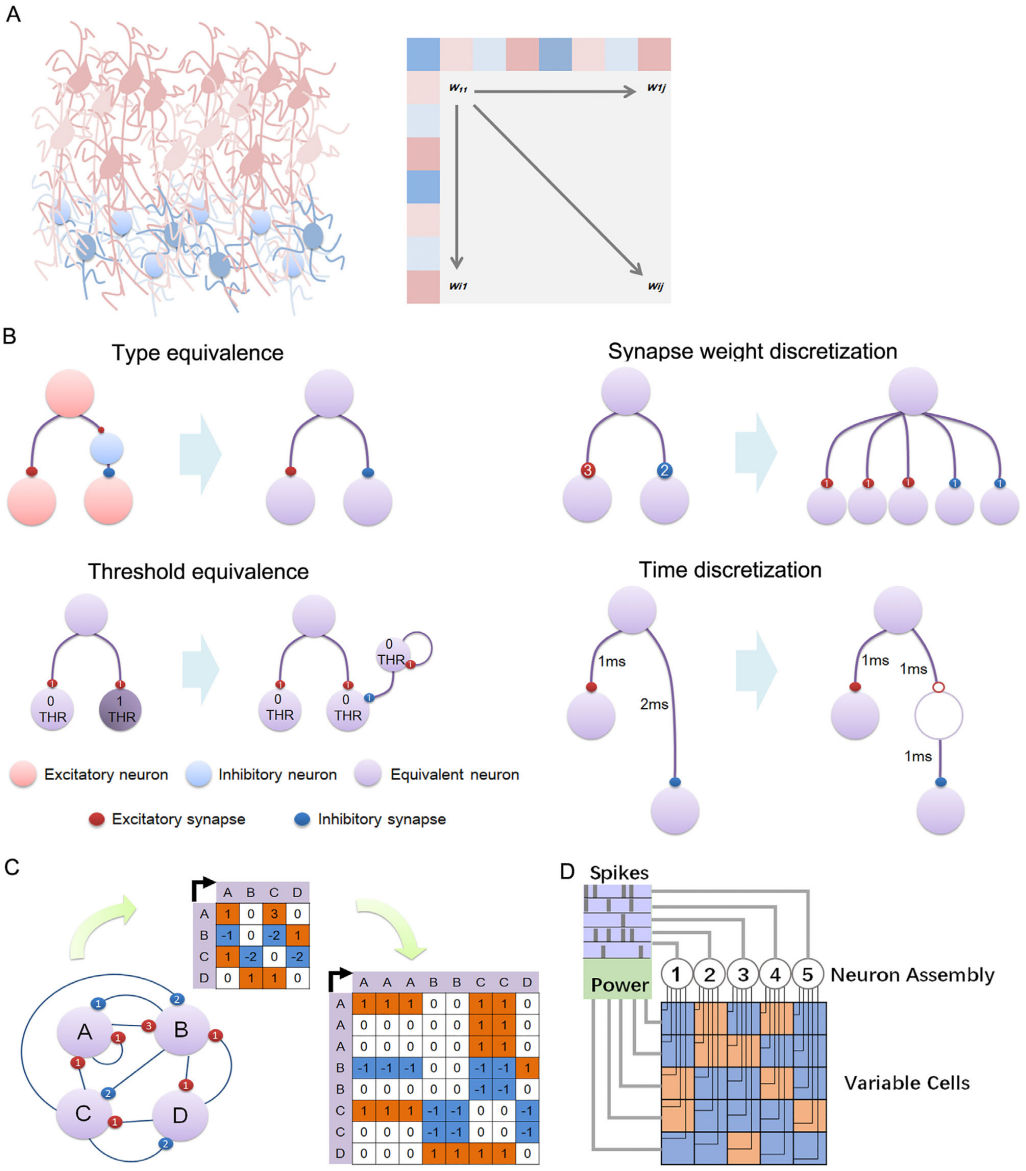
Mathematical model transformation from real to neurobionic assembly. A, Illustration of a connection matrix from a neural assembly. B, Two equivalence and discretization methods for the connection pattern. C, Illustration of the equivalence and discretization for a connection matrix. D, Architecture of an implementable neurobionic device

First, a real neuro‐network can be imagined as a directed matrix (Figure 1A). The weight of each synapse *Wij* refers to the *i* to *j* connection. Pyramidal neurons are a kind of excitatory neurons, while interneurons are a kind of inhibitory neurons.^18^, ^19^ Therefore, in Figure 1A, each row has a consistent sign of the weight. In our mathematical model, we plan to break this limitation by type equivalence, which means that an inhibitory neuron will be transformed into synapses that directly inhibit the next set of neurons (Figure 1B). By this equivalence, every neuron in the matrix is similar, having both excitatory and inhibitory synapses. The next step is to achieve threshold equivalence to unitize each neuron's threshold of activation to zero (Figure 1B). Then, synapse weight discretization is performed to unitize *Wij to* −1, 0, or 1, depending on the weight situation. Finally, the interval from the signal transmission between neurons is also unitized, using a medium neuron to cause a delay. Figure 1C is a given network that is expressed by a directed matrix and then transformed into an equivalent simplified matrix. In theory, this equivalent simplified matrix can describe any kind of complex network. Also, this model has the maximal freedom, which will allow any possible behavior of a natural network to be revealed. Thus, we call this mathematical model a differential of the neuro‐network. By simplification of the neural assembly, we can connect those neurons in an artificial material that consists of small variable cells that can apply positive or negative current to a neuron. The existence of the positive or negative current is decided by the previous active neuron, which is similar to the excitatory/inhibitory synapse. The running speed of the assembly depends on the current circle interval delay. All the variable cells can be modified to a positive or negative mode freely as needed (Figure 1D).

### 2.2 Operation rules of the neurobionic material

The dynamics of the neural network can be expressed by the following recursive formulas:

(1) $$M=\left(\begin{array}{ccc} m_{11} & \cdots & m_{1l}\\ \vdots & \ddots & \vdots \\ m_{k1} & \cdots & m_{kl} \end{array}\right),k=l,\;m_{kl}\in \left\{-1,1\right\},$$

(2) $$S\left(t\right)=\left(Y_{1}^{t}\;\;Y_{2}^{t}\;\ldots Y_{i}^{t}\;\right),\;Y_{i}^{t}\in \left\{0,1\right\},$$

(3) $$X=S\left(t-1\right)\times M,$$

(4) $$Y_{i}^{t}=F\left[X_{i}^{t}\right]=\left\{\begin{array}{c} 0,\;\mathrm{when}\;X_{i}^{t}\leq 0\\ 1,\;\mathrm{when}\;X_{i}^{t}>0 \end{array}\right.,$$

(5) $$S\left(t+1\right)=F\left[S\left(t\right)\times M\right]=\left(Y_{1}^{t+1}\;Y_{2}^{t+1}\;\ldots Y_{i}^{t+1}\;\right).$$

*M* is the equivalent simplified matrix mentioned above. *S*(*t*) is the state of the present network, assembled by *Y_i_^t^*, which denotes the activation (1) or silence (0) of one neuron. *X* is the medium process before judgment is carried out by the function *F*, which determines its activation or silence at the next time point. Although we now have a limited prediction of the behavior or dynamics of this equation set, it is worth noting that the issue we are concerned with is not one state of the *Y_i_^t^* set but the distributions of *Y_i_^t^* over time, which are known as spikes. The initial state of the network is not to be set very exactingly, a single firing neuron is usually chosen. All the simulation experiments were performed by MATLAB v2014. Parameters in each test were available in related sections. Detailed proofs for equations demonstrated in the results are as follows.

### 2.3 Spike probability distribution in a closed neurobionic material assembly

Because the ensemble $X_{i}^{t}$ and $Y_{i}^{t}$ is limited by a circle *T*, for a large number of dimensions *i* as well as iterations, $X_{i}^{t}$ will form a distribution. For each $X_{i}^{t}$, $\bar{X_{i}^{t}}$ can be calculated by $\sum_{1}^{T}\frac{X_{i}^{t}}{T}$. During the iterations, the probability of $Y_{i}^{t}=\;0\;\mathrm{or}\;1$ can also be calculated as $P_{i}^{0}\;\mathrm{or}\;P_{i}^{1}$. The relationship between $\bar{X_{i}^{t}}$ and $P_{i}^{1}\left(\mathrm{or}\;P_{i}^{0}\right)$ is expressed as follows.

By enough iterations, that is, t→∞, $\bar{X_{i}^{t}}$ will be fixed as $\bar{X_{i}}$, having less of a relationship with *t*. Define an ensemble $X\;=\;\{\bar{X_{1}}\;\bar{X_{2}}\cdots \bar{X_{i}}\}$. Because $Y_{i}^{t}=\;0\;\mathrm{or}\;1$ is similar to some kind of sampling process from ensemble X, each distribution can be considered a normal distribution by a sufficiently large *i* and *t* as:

(6) $$P\left(\bar{X}|Y_{i}^{t}=1\right)∼N\left(u_{1},\sigma^{2}\right),$$

(7) $$P\left(\bar{X}|Y_{i}^{t}=0\right)∼N\left(u_{0},\sigma^{2}\right).$$

According to Bayes’ theorem,

(8) $$P\left(Y_{i}^{t}=1|\bar{X}\right)=\frac{P\left(\bar{X}|Y_{i}^{t}=1\right)\cdot P\left(Y_{i}^{t}=1\right)}{P\left(\bar{X}\right)},$$

(9) $$P\left(Y_{i}^{t}=0|\bar{X}\right)=\frac{P\left(\bar{X}|Y_{i}^{t}=0\right)\cdot P\left(Y_{i}^{t}=0\right)}{P\left(\bar{X}\right)}.$$

Therefore,

$$\begin{array}{c} \log\frac{P\left(Y_{i}^{t}=1|\bar{X}\right)}{P\left(Y_{i}^{t}=0|\bar{X}\right)}=\log\frac{P\left(\bar{X}|Y_{i}^{t}=1\right).P\left(Y_{i}^{t}=1\right)}{P\left(\bar{X}|Y_{i}^{t}=0\right).P\left(Y_{i}^{t}=0\right)}\\ \;=\log P\left(\bar{X}|Y_{i}^{t}=1\right)-\log P\left(\bar{X}|Y_{i}^{t}=0\right)+\log\frac{P\left(Y_{i}^{t}=1\right)}{P\left(Y_{i}^{t}=0\right)}\\ \;=-\frac{{\left(\bar{X}-u_{1}\right)}^{2}}{2\sigma^{2}}+\frac{{\left(\bar{X}-u_{0}\right)}^{2}}{2\sigma^{2}}+\log\frac{P\left(Y_{i}^{t}=1\right)}{P\left(Y_{i}^{t}=0\right)}\\ \;=\frac{2\left(u_{1}-u_{0}\right)\bar{X}+\left({u_{0}}^{2}-{u_{1}}^{2}\right)}{2\sigma^{2}}+\log\frac{P\left(Y_{i}^{t}=1\right)}{P\left(Y_{i}^{t}=0\right)}. \end{array}$$

Thus,

$$\begin{array}{ccc} & & \frac{P\left(Y_{i}^{t}=1|\bar{X}\right)}{P\left(Y_{i}^{t}=0|\bar{X}\right)}=\\ & & \;\frac{P\left(Y_{i}^{t}=1|\bar{X}\right)}{1-P\left(Y_{i}^{t}=1|\bar{X}\right)}=e^{\frac{2\left(u_{1}-u_{0}\right)\bar{X}+\left({u_{0}}^{2}-{u_{1}}^{2}\right)}{2\sigma^{2}}+\log\frac{P\left(Y_{i}^{t}=1\right)}{P\left(Y_{i}^{t}=0\right)}}. \end{array}$$

Hence,

(10) $$\begin{array}{ccc} P\left(Y_{i}^{t}=1|\bar{X}\right) & = & \frac{1}{1+e^{\frac{\left(u_{0}-u_{1}\right)\bar{X}}{\sigma^{2}}+\frac{\left({u_{1}}^{2}-{u_{0}}^{2}\right)}{2\sigma^{2}}+\log\frac{P\left(Y_{i}^{t}=0\right)}{P\left(Y_{i}^{t}=1\right)}}}\\ & & ∼\;\frac{1}{1+e^{A\bar{X}+C}}. \end{array}$$

Clearly, *A* < 0 and *C* is a constant. $P(\;Y_{i}^{t}=\;1\bar{X})$ conforms to a logistic curve.

Therefore, the relationship between $\bar{X_{i}^{t}}$ and $P_{i}^{1}$ is $P_{i}^{1}=\frac{1}{1+e^{A\bar{X}+C}}$.

In addition,

$$\begin{array}{c} \bar{X_{j}^{t}}=\frac{\sum_{1}^{T}X_{j}^{t}}{T}=\frac{\sum_{1}^{T}\sum_{1}^{i}\left(Y_{i}^{t}\times m_{ij}\right)}{T}=\frac{\sum_{1}^{T}\sum_{1}^{i}Y_{i}^{t}}{T}\\ \;\times \;m_{ij}=\frac{\sum_{1}^{i}\sum_{1}^{T}Y_{i}^{t}}{T}\times m_{ij}\\ \;=\sum_{1}^{i}P_{i}^{1}\times m_{i}j=\left(P_{1}^{1}P_{2}^{1}\cdots P_{i}^{1}\right)\times M. \end{array}$$

Substituting this function into Equation (10) yields

(11) $$\begin{array}{c} \left(\;P_{1}^{1}\;\cdots P_{i}^{1}\right)\times M=\frac{1}{A}.\left(\log\frac{1-P_{1}^{1}}{P_{1}^{1}}-C\cdots \log\frac{1-P_{i}^{1}}{P_{i}^{1}}-C\;\right). \end{array}$$

### 2.4 Shannon entropy shifts under stimulation in the neurobionic material

It is not difficult to understand that the number of $Y_{i}^{t}$ sets is finite, relying on the previous state of the network, known as S(t−1), meaning that the state of the network is a fixed point or a loop.

If the state of S(t) is a loop, we can begin on any point as (0)→S(1)→S(2)⋯→S(t)→S(0)⋯, and the cycle is T. Assume that cycle T is large enough and that S(t) is unpredictable for an observer, such as a series of random systemic states. According to the Boltzmann entropy theorem, the entropy for the assembly can be calculated as

$$S_{T}=k\ln T.$$

On the other hand, we can also calculate the entropy of the assembly for each neuron by its spike probability $P_{i}$. According to the Shannon entropy theorem,

$$S_{P}=-\sum_{1}^{i}P_{i}\log P_{i}.$$

(To be more precise, because the amount of $S_{T}$ is not exactly random but restricted by the dynamic rules, namely, $S_{T}\leq S_{P}$)

$$k\ln T=-\sum_{1}^{i}P_{i}\log P_{i}.$$

Imaging there are N neurons firing as described above, and n neurons are then restricted ($P{}_{1}^{\prime }\cdots \;P{}_{n}^{\prime }=\;0\;\mathrm{or}\;1$). The new circle $T^{\prime }\leq T$.

$$k\ln T^{\prime }=-\sum_{n+1}^{i}P_{i}^{\prime }\log P_{i}^{\prime }.$$

Then,

$$\begin{array}{ccc} -\sum_{n+1}^{i}P_{i}^{\prime }\log P_{i}^{\prime } & = & k\ln T^{\prime }\leq k\ln2^{N-n}\leq k\ln2^{N}\\ & & -\;n=k\ln T-n\leq -\sum_{n+1}^{i}P_{i}\log P_{i}. \end{array}$$

That means the spike probabilities of left neurons are polarized.

### 2.5 Stability of neurobionic material relates to excitatory/inhibitory proportions

Assume that there is an iteration system described by functions given by Equations (1)‐(5). For matrix given in Equations (1), we can set the parameters $E_{-1}$ and $E_{1}$, as well as $N^{2}$, representing the expected proportion of −1 or 1, and the number of elements ($E_{-1}+\;E_{1}=\;1$).

As mentioned above, when t→∞, vector $S\left(t\right)\;=\left(Y_{1}^{t}\;\;Y_{2}^{t}\;\ldots Y_{i}^{t}\;\right)$ will be trapped in a circle *T* ($Y_{i}^{t}\in \left\{0,1\right\}$). The probability of $Y_{i}^{t}=\;1(P_{i}^{1}$) is the solution of the nonlinear system of Equation (11):

(11) $$\left(P_{1}^{1}\;\cdots P_{i}^{1}\right)\times M=\frac{1}{A}.\left(\;\log\frac{1-P_{1}^{1}}{P_{1}^{1}}-C\cdots \log\frac{1-P_{i}^{1}}{P_{i}^{1}}-C\;\right).$$

Let us define a value $\tilde{N}$, which denotes the expected amount of $Y_{i}^{t}=\;1$ for each *t*. Here, we doubt that $\tilde{N}$ is stable and consider how to calculate $\tilde{N}$. The proof is as follows.

Suppose that at one time *t*, the amount of $Y_{i}^{t}=\;1$ is N(t), and N(t)<N. Substituting Equation (3) into Equation (4), the value of $Y_{i}^{t+1}$ is determined by

$$\begin{array}{ccc} Y_{i}^{t+1} & = & F\left[X_{i}^{t+1}\right]\\ & = & \left\{\begin{array}{c} 0,\;\mathrm{when}\;\left(Y_{1}^{t}\;\;Y_{2}^{t}\;\cdots Y_{i}^{t}\;\right)\times \left(\begin{array}{c} M_{1i}\\ \vdots \\ M_{\mathrm{ki}} \end{array}\right)\leq 0\\ 1,\;\mathrm{when}\;\left(Y_{1}^{t}\;\;Y_{2}^{t}\;\cdots Y_{i}^{t}\;\right)\times \left(\begin{array}{c} M_{1i}\\ \vdots \\ M_{\mathrm{ki}} \end{array}\right)>0 \end{array},\;i=k.\right. \end{array}$$

Taking the parameters $E_{-1}$ and $E_{1}$ into consideration, we can easily see that when $E_{-1}<E_{1}$, the probability of $Y_{i}^{t+1}=\;1$ is higher than the situation $E_{-1}>E_{1}$. This is because $\left(Y_{1}^{t}\;\;Y_{2}^{t}\;\ldots Y_{i}^{t}\;\right)\times \left(\begin{array}{c} M_{1i}\\ \vdots \\ M_{ki} \end{array}\right)$ is similar to a random sampling process from $\left(\begin{array}{c} M_{1i}\\ \vdots \\ M_{ki} \end{array}\right)$. $Y_{i}^{t}=\;1$ means that $M_{ki}$ is selected and summed into $X_{i}^{t+1}$.

Therefore, the value of $X_{i}^{t+1}$ is similar to the binomial distribution

(12) $$P\left(X_{i}^{t+1}\;\right)=\sum_{n=0}^{N\left(t\right)}\left[\begin{array}{c} N\left(t\right)\\ k \end{array}\right]{E_{1}}^{n}{E_{-1}}^{N\left(t\right)-n}.$$

When *N* is sufficiently large, $E_{-1}\;\mathrm{and}\;E_{1}$ are not very different, and the binomial distribution will approximate a normal distribution:

(13) $$P\left(X_{i}^{t+1}\;\right)∼N\left(u=N\left(t\right)\cdot E_{1},\;\sigma^{2}=N\left(t\right)\cdot E_{1}\cdot \;E_{-1}\right).$$

Therefore, the probability of $P(X_{i}^{t+1}>0)$ is equal to the difference in the cumulative density function:

$$\begin{array}{ccc} P\left(X_{i}^{t+1}>0\right) & = & \Phi \left(\frac{N\left(t\right)-N\left(t\right)\cdot \;E_{1}}{\sqrt{N\left(t\right)\cdot E_{1}\cdot E_{-1}}}\right)\\ & & -\;\Phi \left(\frac{\frac{N\left(t\right)}{2}-N\left(t\right)\cdot E_{1}}{\sqrt{N\left(t\right)\cdot E_{1}\cdot E_{-1}}}\right). \end{array}$$

If N(t) is stable, $N\;\left(t+1\right)=\;N\times P\;\left(X_{i}^{t+1}>0\right)=\;N\left(t\right)$.

The value of N(t) is the solution to the function

(14) $$\begin{array}{ccc} N\left(t\right) & = & N\cdot \left[\Phi \left(\frac{N\left(t\right)-N\left(t\right)\cdot E_{1}}{\sqrt{N\left(t\right)\cdot E_{1}\cdot E_{-1}}}\right)\right.\\ & & -\;\left.\Phi \left(\frac{\frac{N\left(t\right)}{2}-N\left(t\right)\cdot E_{1}}{\sqrt{N\left(t\right)\cdot E_{1}\cdot E_{-1}}}\right)\right]. \end{array}$$

When $E_{1}$ is within a certain range, N(t) is similar to a fixed point.

### 2.6 In vivo experiments

#### 2.6.1 Animals and grouping

Eighteen male Sprague‐Dawley (SD) rats weighing 200‐250 g were used in the experiments. All rats were raised and maintained in a specific pathogen‐free (SPF)‐II environment. All operative procedures were performed under strict aseptic conditions. Before experimentation, the rats were allowed to acclimate to laboratory conditions. All animal experiments were in accordance with the ARRIVE guidelines. All experiments were approved by the Animal Care and Use Committee of Huashan Hospital, Fudan University.

As we calculated in the neurobionic tissue model, the proportion of inhibitory neurons and the stimulation intensity will affect the amount of active neurons. So we planned to use in vivo brain tissue to verify the prediction. The brain cortex tissue diversity was created by the ferric chloride (FeCl_3_) injection (high inhibitory proportion and high stimulation intensity) and FeCl_3_ + desferrioxamine (DFO) treatment (medium inhibitory proportion and medium stimulation intensity). Thus, the rats were divided into three groups: Tissue A: the sham control, with intracortical injection of saline only (low inhibitory proportion and low stimulation intensity, n = 6); Tissue C: FeCl_3_‐induced group, with intracortical injection of freshly prepared FeCl_3_ solution (100 mM, 5 μL) and subsequent treatment with saline (10 mL/kg) for 14 days (n = 6); Tissue B: with intracortical injection of freshly prepared FeCl_3_ solution (100 mM, 5 μL) and subsequently treated with DFO (100 mg/kg, concentration: 10 mg/mL for 14 days, n = 6). No unexpected death existed during the experiment.

#### 2.6.2 FeCl_3_‐induced epileptic model

After a midline scalp incision, the pericranial muscles and fascia were retracted laterally. Next, a bone hole (1 mm diameter) was drilled 1 mm posterior and 2 mm lateral to the bregma (the bone hole locates at the locus of the sensorimotor cortex following the coordinates of the stereotaxic atlas). Through this hole, 5 μL of freshly prepared aqueous solution containing 100 mM FeCl_3_ was injected over a period of 5 minutes into the sensorimotor cortex at a depth of 0.5 mm using a microinjection syringe. The needle was held in place for an additional 30‐60 seconds to prevent reflux. For rats that would undergo electroencephalograph (EEG) recording, a bipolar electrode was implanted, one on the surface of the sensorimotor cortex under the hole used for FeCl_3_ injection, and another reference electrode was simultaneously implanted on the contralateral frontal dura surface. Subsequently, the bone hole was covered with bone wax, and the wound was closed with stitches. After modeling, rats were randomly grouped into groups. EEG was recorded from days 1 to 28 after intracortical injection of FeCl_3_. In the sham control animals (Tissue A), 5 μL of saline was injected intracortically into the sensorimotor cortex.

#### 2.6.3 EEG study

Wireless EEG‐collecting implants (DSI, USA) were used to record EEG of rats 24 hours per day for 28 days (sampling rate 200 Hz). Each EEG file was analyzed manually by scanning through the EEG recording on the computer screen. Average field potential was calculated by Clampfit 9.0 program. The investigators were blinded to the animal group assignment.

#### 2.6.4 Western blot analysis

The cortex tissue of the injection area was collected for further analysis. All of the nuclear and cytoplasmic proteins (100 μg) of the brain tissue were size‐fractionated using SDS‐polyacrylamide gel electrophoresis (SDS‐PAGE) and transferred to Immobilon‐P membranes (Millipore). The blotted membranes were incubated with primary antibodies against GABAR‐1/2, GAPDH (Abcam, HK Ltd), followed by incubation with an HRP‐conjugated secondary antibody (Jackson). The immunoreactivity was detected using an enhanced chemiluminescence reaction system (Amersham Pharmacia Biotech).

#### 2.6.5 Immunofluorescent staining

Brain tissues were fixed in 10% formalin, embedded in paraffinor paraffin, sectioned at 4 μm thicknesses, and stained with antiparvalbumin (rabbit polyclone, Abcam, HK Ltd). The secondary antibody was goat anti‐rabbit immunoglobulin. Nuclei were visualized with 4′,6‐diamidino‐2‐phenylindole (DAPI). Slides were photographed for red (Alexa Fluor 594) and green (Alexa Fluor 488) fluorescence with a fluorescent microscope (E400; Nikon, Tokyo, Japan). Cells were analyzed as the amount of positive staining per field (×200) in the area of injection site. Five random areas in three sections from each brain were chosen for calculation, which was performed by 2 independent observers who were blinded to group assignment.

#### 2.6.6 Statistical analysis

Data of behavioral, EEG, Western blot, and tissue analysis were all analyzed by two independent researchers who were blinded to each group. All the data are presented as the means ± SD. Data were analyzed using independent‐sample *t*‐tests and one‐way analysis of variance (ANOVA). *P*‐value < .05 was considered as statistically significant. All statistical analyses were performed using Graphpad Prism 5.0 for Windows.

## 3 RESULTS

### 3.1 General features of the neurobionic tissue model

The neurobionic material comprises the artificial neurons, the modifiable connective chip matrix driven by the power system. The output of neurobionic material is characterized by spike frequency of each neuron. If there is no interference with this assembly, it is not difficult to understand that the amount of the *Y_i_^t^* set is finite, relying on the previous state of the network, known as S(t−1), meaning the state of the assembly is a fixed point or a loop.

In a closed neurobionic material assembly constructed by random connections, we have already proved that the spike probability distribution of each neuron is in accordance with a sigmoid curve in the assembly (see Materials and Methods). In the rest of the stable state, total assembly activity is related to the proportion of excitatory synapses. The spike frequency or probability ($P_{i}$) of a certain neuron possesses a weak correlation with the expectative accumulation calculated by the connection matrix, but has a close relation to the posterior average input accumulation$\;\bar{X_{i}}$. The relationship can be expressed as the following equation:

(10) $$P_{i}=\frac{1}{1+e^{A\bar{X_{i}}+C}}.$$

**FIGURE 2:**
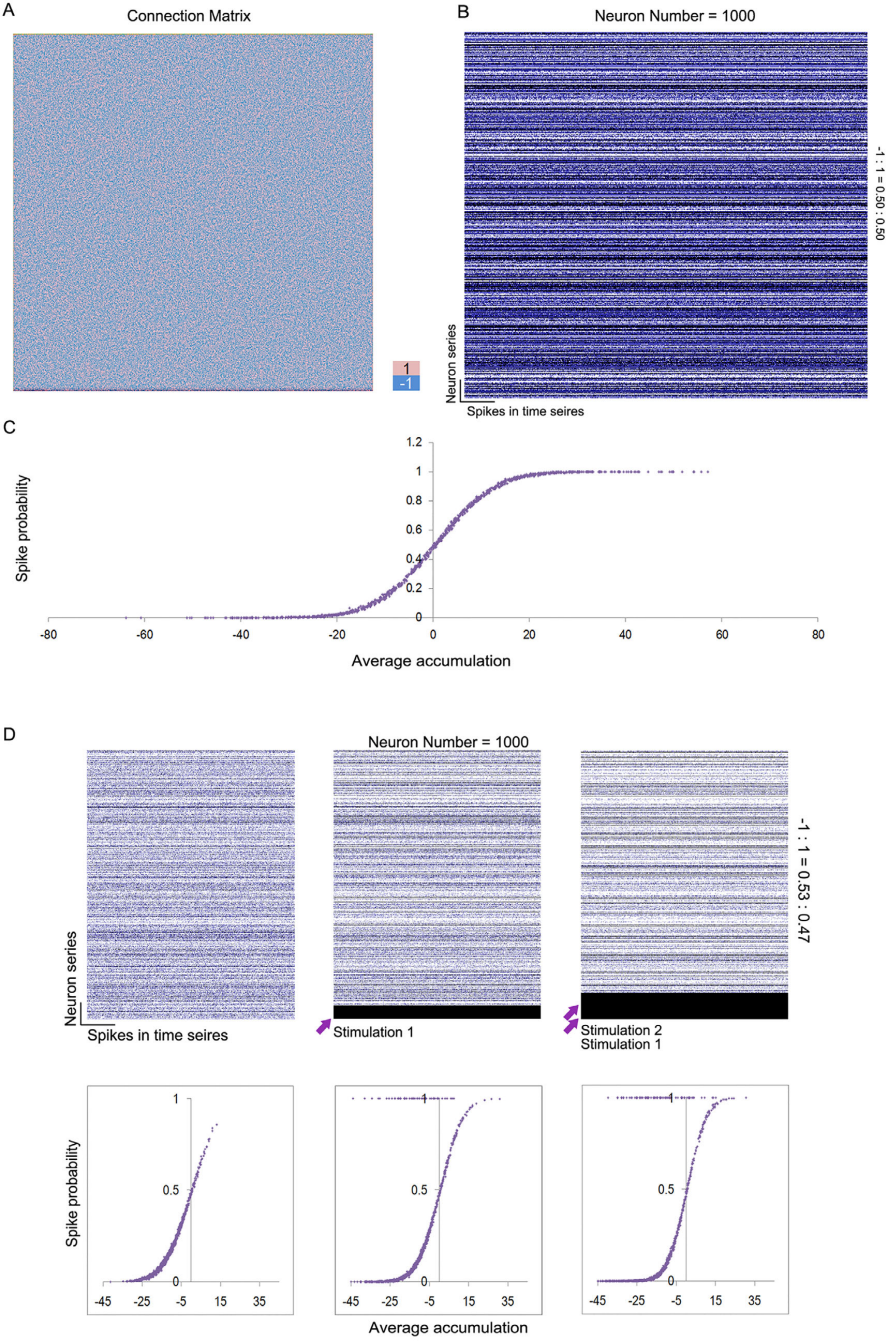
Spontaneous spike probability distribution of a closed neurobionic assembly. A, Random connection matrix with similar excitatory/inhibitory proportions. Neuron number = 1000, excitatory/inhibitory = 1:1. B, Spike‐time series of each neuron in the assembly. Each dot represents a firing. C, Average accumulation‐spike probability curve of the neurobionic material assembly. D, Spike‐time series and related average accumulation‐spike probability curve of the neurobionic material assembly, with or without stimulation inputs. Neuron number = 1000, excitatory/inhibitory ratio = 0.53:0.47. Arrows: neurons of persistent activation; stimulation 1 = stimulation 2 = 50 neurons

$P_{i}$ is the spike probability, $X_{i}$ is the expected accumulated input, and *A* and *C* are network‐related parameters. This basic equation describes the relationship between the spike probability and average input accumulation for a neuron. In Figure 2A, we demonstrate a representative example of a random connective neural assembly of 1000 neurons with similar amounts of excitatory and inhibitory synapses. Spikes in the time series for each neuron, which have a fixed activation probability without any regularity of distribution, are shown in Figure 2B. Taking average accumulation (average number of input signals at each time point) into consideration, the relationship presents a sigmoid curve (logistic function, Figure 2C). It is noteworthy that this is a closed assembly without any effect, and the sigmoid curve is automatically formed by a proper excitatory/inhibitory connection proportion. In given situations, as the inhibitory proportion exceeds the upper limit, the closed assembly cannot operate continuously. The estimated highest inhibitory proportion will be calculated below. If we input some stimulations into this network, such as a persistent activation of a portion of neurons, the firing rates of almost all of the rest of neurons will change. However, the sigmoid distribution will be maintained except for the alteration of parameters *A* and *C* (Figure 2D).

### 3.2 Spike probabilities in the neurobionic material can meet the solution of a nonlinear equation set

**FIGURE 3:**
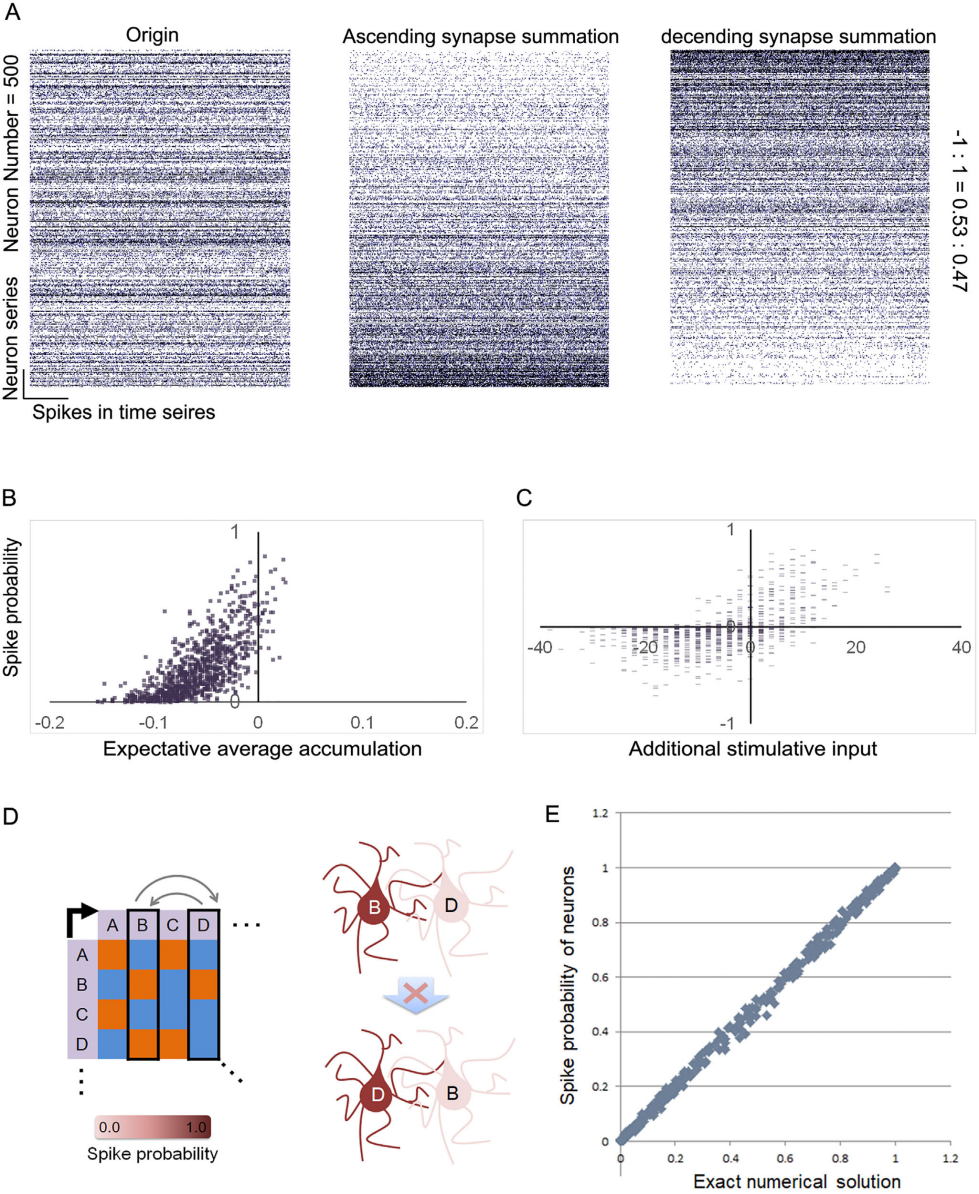
Spike probabilities cannot be predicted by linear operations. A, Ranking by ascending or descending synapse weight summation cannot sort the spike probabilities. Neuron number = 500, excitatory/inhibitory = 0.53:0.47. B, Weak correlation between the actual spike probability and the expected probability calculated by the synapse weight summation. C, Weak correlation between the spike probability shift and the expected shift calculated by the input summation. D, Swapping the column of the connection matrix cannot maintain the previous spike probability. E, Scatter diagram between exact numerical solution and related spike probability. Neuron number = 400, excitatory/inhibitory ratio = 0.5:0.5, *R* = 0.98

Activation pattern of each neuron is usually decided by the connection pattern of the network, which is defined as the directed connection matrix *M*, as mentioned above. However, it is impossible to predict the spike probability of each neuron directly by *M*. The reason is that the spike probability is the solution of a set of nonlinear equations and has been proved (see Materials and Methods). In particular, we cannot calculate the spike probability by summing the synapses weights toward one neuron in *M*, even for the expectation. In Figure 3A, we employed a closed neuron cluster of 500 neurons, and the left panel shows the origin activation pattern of each neuron. However, when we adjust matrix *M* by summing the total number of synapses weights connected to each neuron and then array them in ascending or descending order, the spike probability is not strictly arranged by similar order, despite the general trend. In a 1000‐neuron closed neuron cluster, the spike probability of each neuron has a weak linear correlation with the expected accumulation from the assembly (Figure 3B). In addition, when stimulants exist, the strength of continuous stimulation relative to one neuron has a weak correlation with its spike probability shift (Figure 3C). That is to say, with a stepwise rise in persistent input to a certain neuron, the spike probability cannot be elevated in a consistent manner. In most situations, the activation pattern will not change synchronously only by swapping the row in *M* (Figure 3D). The entire phenomenon mentioned above indicates that the spike probability of one neuron in the assembly is not a solution of a simple or linear equation. The following is an equation set that describes their relationship in a steady state (see Materials and Methods):

(11) $$\left(\;P_{1}^{1}\cdots P_{i}^{1}\;\right)\times M=\frac{1}{A}.\left(\;\log\frac{1-P_{1}^{1}}{P_{1}^{1}}-C\cdots \log\frac{1-P_{i}^{1}}{P_{i}^{1}}-C\;\right).$$

In this equation set, $S_{i}$ is the summation of external stimulations to one neuron in the neurobionic material. If there is no stimulant, $S_{i}$ equals zero. To verify this finding, we give a random equation set mentioned above and solve it by MATLAB. Then we use the same parameters to operate the neural assembly to a steady state, and count the spike probability of each neuron. Finally, we plot the scatter diagram between exact numerical solution and related spike probability, and find they are linear dependent exactly (Figure 3E, *R* = 0.98).

### 3.3 Distribution shift of the spike probability under stimulation

**FIGURE 4:**
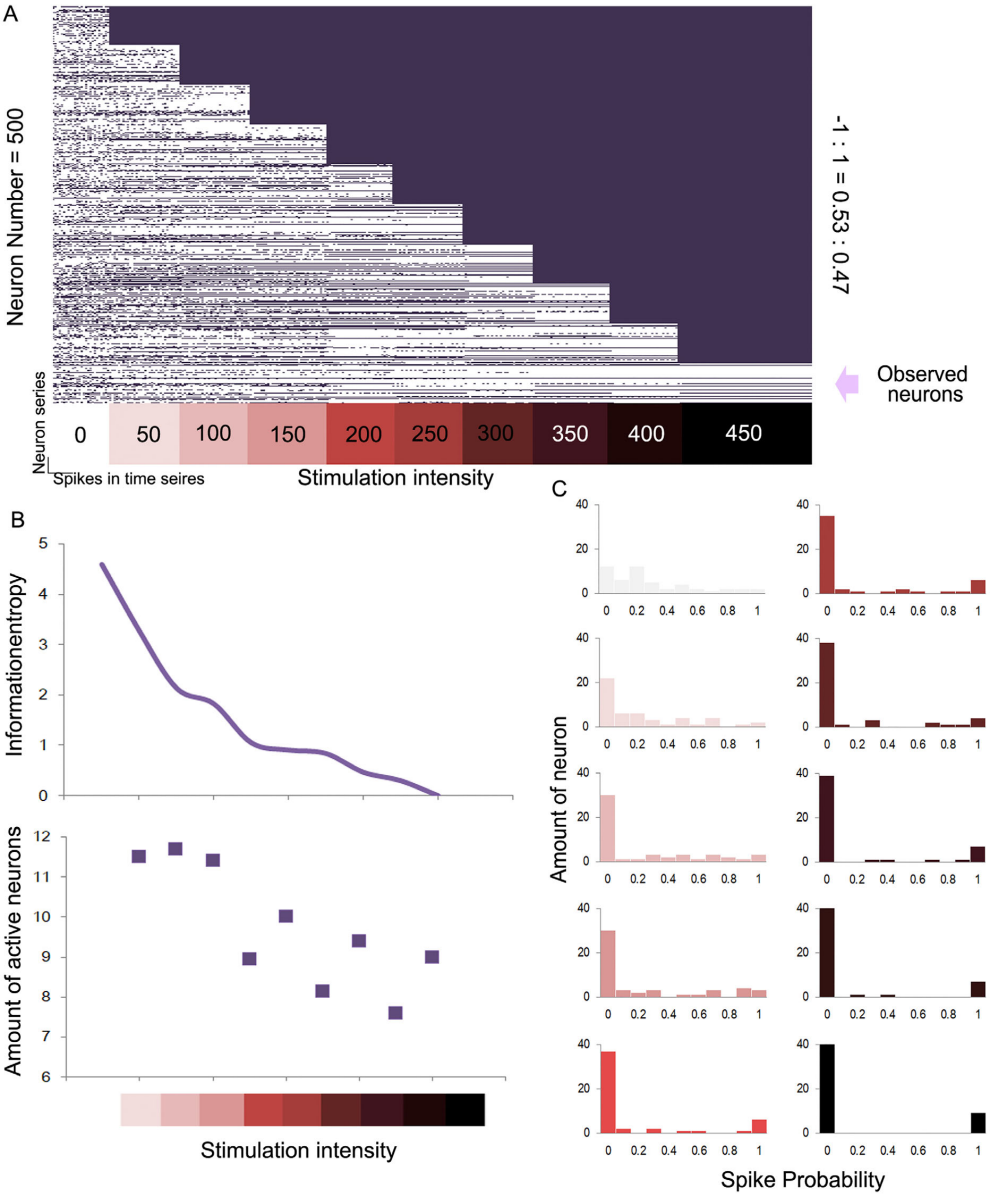
Polarization of the spike probability resulting from an increase in stimulations. A, Spike‐time series of each neuron in the neurobionic material, affected by increases in the stimulations. Neuron number = 500, excitatory/inhibitory ratio = 0.53:0.47. B, Information entropy of the observed neuron spike probabilities from the increase in stimulations. C, Polarization of the spike probability owing to the increase in stimulations

To date, we have described only the dynamic patterns in a closed neurobionic material assembly. In a changing environment, the assembly will be affected by inputs at every moment, typically leading to a more certain state. We have already proved that in a neural assembly, increasing the stimulation strength will elevate the slope of the original sigmoid curve and polarize the spike probabilities of each neuron, leading to a lower Boltzmann entropy (or Shannon entropy, see Materials and Methods). Figure 4A shows a closed assembly that receives inputs step by step. We can see the shift in the spike probabilities of each neuron caused by the increasing input. The entropy as well as the number of active neurons of the whole assembly is decreased at each step (Figure 4B). Some spike probabilities are increased, while others are suppressed, being polarized (Figure 4C). As mentioned above, the spike shifting rules are in accord with the solution of the equation. This phenomenon has an obvious implication, namely the more information we obtain, the more convinced we become regarding a choice.

### 3.4 The running stability of the neurobionic material

**FIGURE 5:**
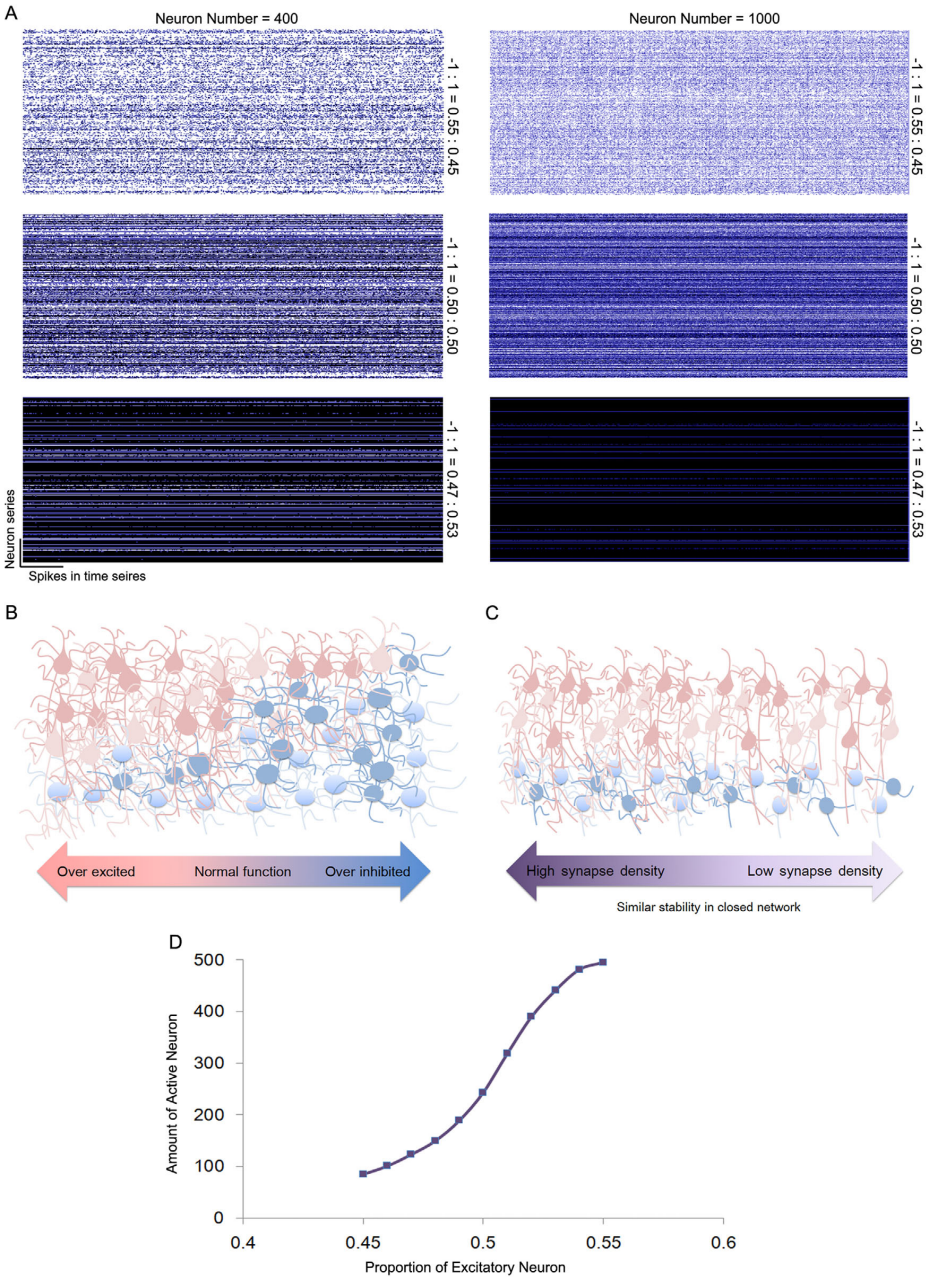
Dependence of the activity of neurobionic assembly on the neuron amount and excitatory/inhibitory proportion. A, Spike‐time series of each neuron in the neurobionic material for different neuron amounts and excitatory/inhibitory proportions. B and C, Illustration of the activity regularity for different parameters. D, Relationship between the excitatory/inhibitory ratio and assembly activity. Neuron number = 500

The running stability of the neurobionic material is of great importance for its functions and materials design. To be adaptive to the changing inputs, the assembly transforms into a certain stable state, meaning that the activation of each neuron causes a switch to a certain mode, being excited or inhibited. In this neurobionic material, the running stability is closely related to the distribution of −1 and 1 in matrix *M*. The simulation test also demonstrates that the number of neurons is another factor affecting the assembly stability. Even the spatial distribution of −1 and 1 is set at random; stability seems to be an intrinsic property under the proper parameters. Figure 5A shows the assembly stability under different parameters. To keep the activation of each neuron stable in a closed neurobionic material assembly, the distribution of −1 and 1 in *M* should be near half and a half to avoid over excitation or inhibition (Figure 5B). As the neuron number increases, the proportion of 1 has to grow in step to remain stable. It is notable that the proportion of 0 in matrix M will not influence the stability in a closed network, which is related to the synapse density (Figure 5C). However, when the assembly is not closed, which means that extra input exists, the network behavior will differ among various synapse densities. Now we have already proved that the total spike rate is related to the number of neurons as well as the proportions of excitatory and inhibitory neurons (see Materials and Methods). The details can be described by Equation (14).

(14) $$\begin{array}{ccc} N\left(t\right) & = & N\cdot \left[\Phi \left(\frac{N\left(t\right)-N\left(t\right)\cdot E_{1}}{\sqrt{N\left(t\right)\cdot E_{1}\cdot E_{-1}}}\right)\right.\\ & & -\;\left.\Phi \left(\frac{\frac{N\left(t\right)}{2}-N\left(t\right)\cdot E_{1}}{\sqrt{N\left(t\right)\cdot E_{1}\cdot E_{-1}}}\right)\right]. \end{array}$$

In this transcendental equation, N(t) means the expected number of active neurons at one time point. N is the total number of neurons, while $E_{1}\;$and $E_{-1}$ are the excitatory and inhibitory neuron proportions. Φ is the accumulation density function for a normal distribution. Figure 5D demonstrates some randomly connected neuron assemblies (500 neurons) with various excitatory neuron proportions, which were in accordance with our prediction.

### 3.5 Mathematical neurobionic model behaves as the real cortex

**FIGURE 6:**
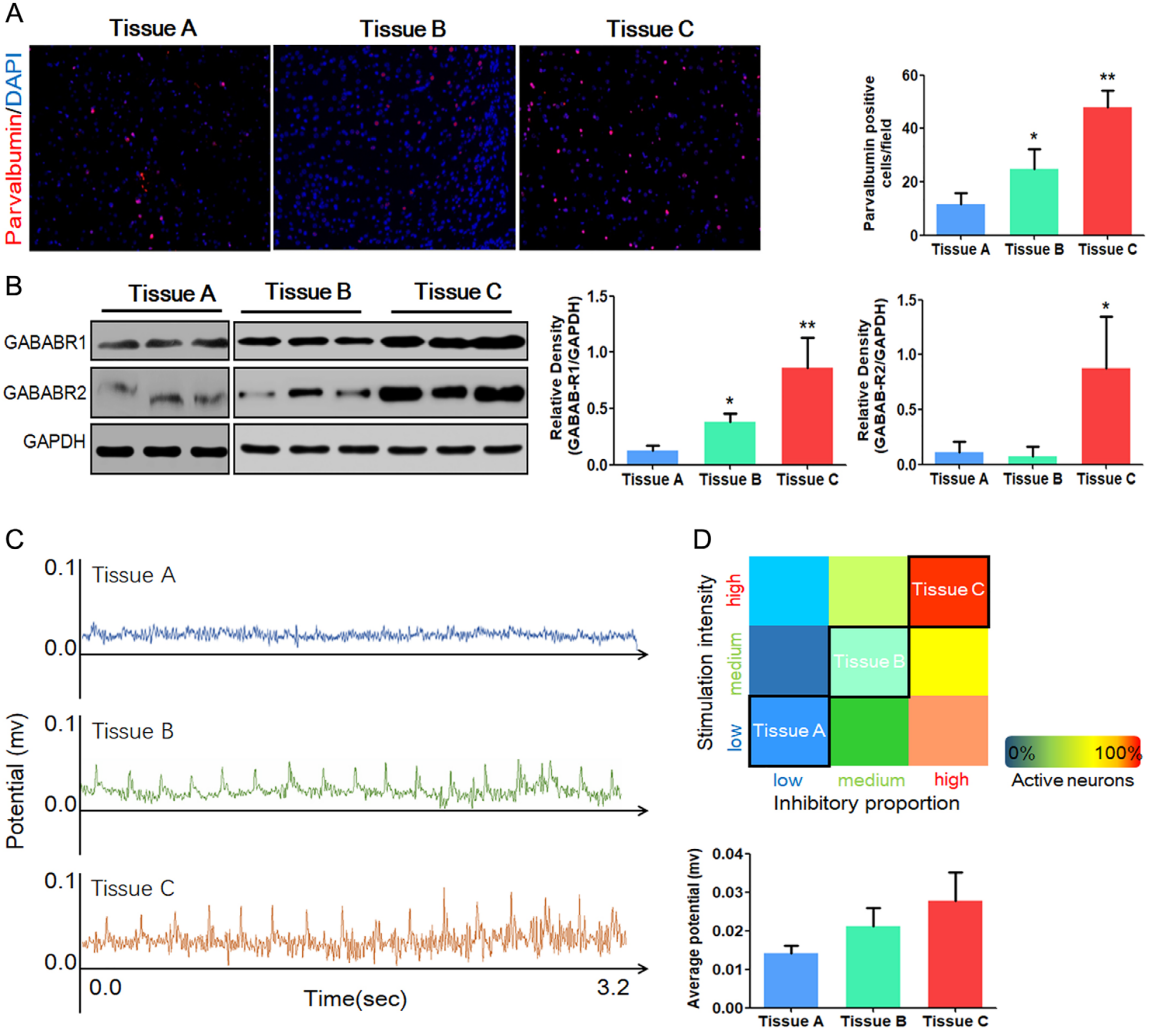
Activation of the neurobionic assembly is similar to the real cortex in vivo. A, Immunofluorescent staining of parvalbumin and 4′,6‐diamidino‐2‐phenylindole (DAPI) in different rat cortex (200×). Red: parvalbumin, blue: DAPI. B, Western blots and histograms showing relative levels of GABAR1 and GABAR2 in Tissues A, B, and C. C, Representative in vivo EEG recording for Tissues A, B, and C cortex. D, Theoretical prediction of the activation in neurobionic tissue. Tissue A: normal cortex; Tissue B: FeCl_3_‐induced injured cortex with DFO intervention; Tissue C: FeCl_3_‐induced injured cortex; n = 6 per group, data are mean ± SD, *P < .05 versus Tissue A, **P < .01 versus Tissue A

Accoding to the results above, the activation of the mathematical neurobionic model depends on the inhibitory neuron proportion and the intensity of stimulation. To verify the consistency with the real cortex, we use a FeCl_3_‐induced injury model to prepare different cortex tissues in vivo followed by EEG recording that can represent the activation. Tissue A is the normal cortex that contains smaller inhibitory neuron proportion and lower intensity of stimulation. Tissue C is the FeCl_3_‐damaged cortex that contains larger inhibitory neuron proportion and higher intensity of stimulation as the epileptogenesis, while Tissue B has an intermediate state between Tissues A and C (Figures 6A and 6B). EEG recording demostrated different average field potentials that represented various activation states among Tissues A, B, and C, which meet the prediction in our neurobionic material model (Figures 6C and 6D).

### 3.6 Phase coupling among neurobionic material assemblies

Since the spike probability of each neuron in an assembly depends only on the connection matrix and stimulant as Equation (11), it seems that memory will be stored only in synapses. However, as mentioned above, the phase of firing will be labeled in the spike sequence after stimulation, making it possible for assemblies coupling to occur.

**FIGURE 7:**
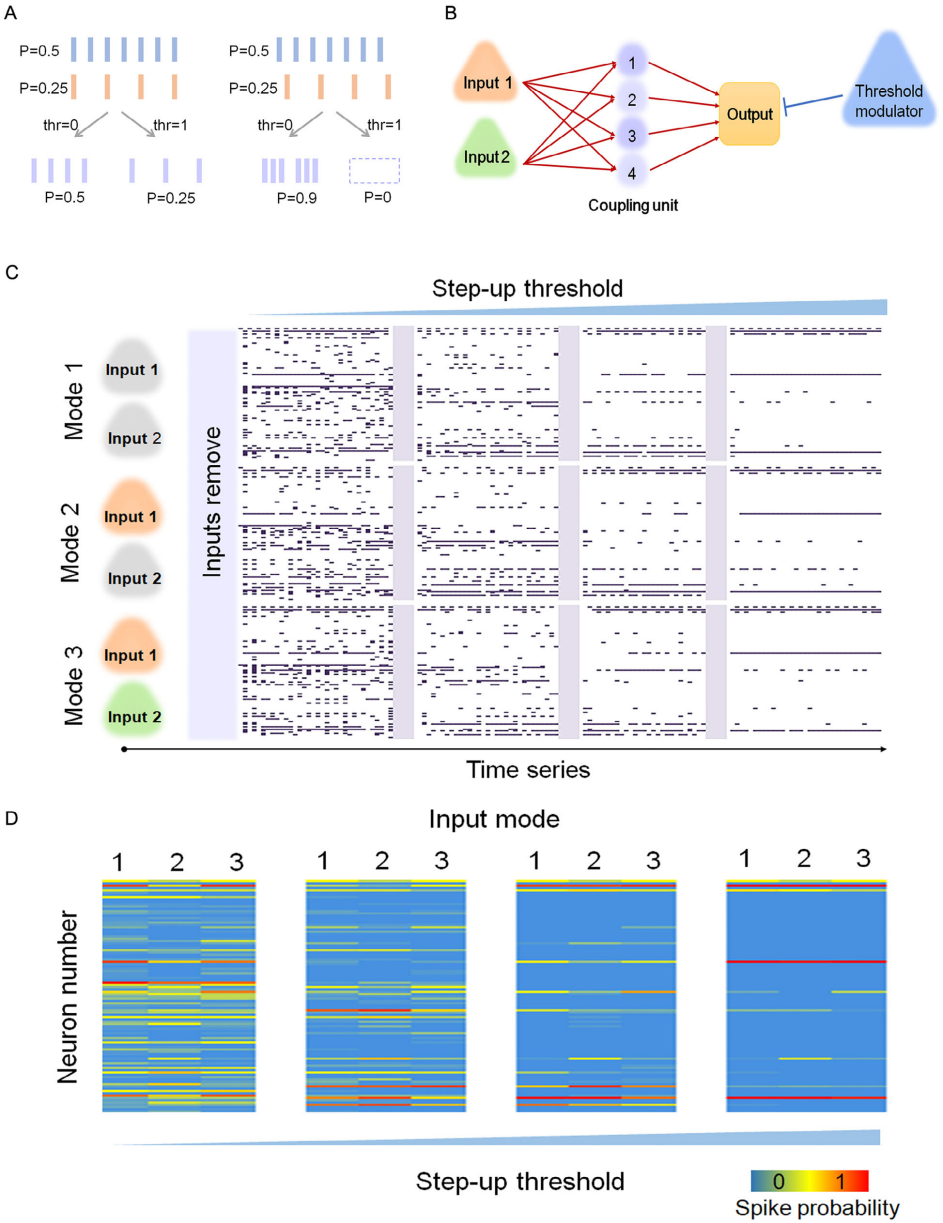
Phase coupling among artificial neurobionic assemblies. A, Different coupling phases and thresholds leading to different outputs from the same two neurons. B, Connection framework of neurobionic assemblies embodying memory. There are two input units and four coupling units to be connected for phase coupling and recording. C, Spike‐time series of the outcome coupled by four coupling units when three kinds of inputs disappear. The threshold of output unit was adjusted by an increasing threshold modulator to highlight firing distinction. D, Heat maps of spike probabilities of outcome unit, which can show three different firing modes representing three kinds of removed inputs. Distinctions remain under an increasing modulation threshold

As in a single neural assembly, both the spike probabilities and relative phase among neurons are decided by the stimulation pattern: hence, neurons in one assembly will not be coupled freely. Figure 7A shows two neurons from different assemblies can achieve free coupling. With .25 and .5 homogeneous spike probabilities, they can couple to a downstream neuron with two major patterns (synchronous or asynchronous). Modulated by different thresholds, the downstream neuron will fire on various probabilities ranging from .0 to .9 (Figure 7A). Figure 7B shows framework neural assemblies that can achieve memorization. The goal of the framework is to distinguish past inputs by modulating the threshold when the inputs are dismissed. First, two different inputs are connected to the coupling units (1 to 4), which are independent of each other, hence making coupling possible. Then, four coupling units are connected to the output unit, which is also unidirectional. As mentioned above, a high or low threshold will lead to different coupling results; thus, there is also a threshold modulator that affects the output signal. Our results show that for different input modes (modes 1, 2, and 3), the output unit can demonstrate obvious distinguishable firing patterns of the unit with the influence of the step‐up threshold (Figures 7C and 7D), representing a recall process of the previous inputs. Here, we focus only on the spike probability combination as a pattern, not the detailed firing sequence. If we do not disturb the coupling units, the phase memory will remain among them, unless the new inputs arrive in and interfere the phase to represent another memory.

## 4 DISCUSSION

Nowadays, artificial electronics can perceive all five natural human senses, including visual, auditory, olfactory, gustatory, and tactile senses.^2^ Moreover, some electronic sensors are somehow beyond human capabilities. For example, although deficient in rendering sharp or high‐resolution images, artificial visual devices have broader optical wavelength and view angle than its biological counterpart.^20^ Besides, sensors for some nonhuman senses, like magnetic field or humidity, are also integrated into artificial sensory organs.^21^ Exoskeleton is wearable device that assists to carry heavy loads or helps paralyzed patients to restore motor function. Controlled by electroencephalogram or electromyogram signal, the lower limb exoskeleton may be a better alternative than a wheelchair.^22^ Instead of basic walking or standing, researchers have developed more complicated functions like running or going up or downstairs.^23^ The fine movement can also be facilitated by hand exoskeleton that helps in patients or elderly people rehabilitation and activities of daily life.^4^ For patients suffering from amputation, new generation of bionic prostheses controlled by EMG signal is emerging, but they are still far from replicating the intrinsic motor functions,^3^ and also the advanced brain functions. Further development of more advanced and functional artificial devices rely on progress on design, material, and fabrication. Many researchers have focused on the improvement of material, while more biocompatible or human‐like design, such as artificial organic synapses, better brain‐machine interfaces or advanced algorithm, are also drawing lot of attention.^1^, ^6^, ^7^

In this work, we proposed a neurobionics material model and found the rules of a spike probability distribution created using a random connection, ultimately inferring an equation set of solutions that can meet the spike probabilities of each neuron. According to our findings, general activity of this neurobionic material is closely related to the excitatory/inhibitory proportion and stimulation intensity, similar to the in vivo situation. Using the frequency or probability of spikes, the neurobionic material can perform operations and offer solutions. This is the intrinsic function of the neurobionic material assembly. By modification of the connective matrix, the function can be transformed to another equation set of different parameters to solve certain problems spontaneously. By this kind of framework, we do not quite understand the real mesoscopic network structure about advanced brain functions, but only adjust the connection parameters according to the equation set.

The spike probability distribution in neurobionic material can be used to solve certain decision‐making problems in certain conditions. Theoretical foundations for modern research on decision making were laid within the development of evidence‐accumulation models.^24^ In vivo experiments revealed that the accumulator value is closely related to the decision probability or firing rate, presented as a sigmoid curve.^25^, ^26^ According to our findings, firing rate distribution in the neurobionic material can spontaneously form a sigmoid curve with or without stimulation, which is the neural basis of decision making. It has already been proved that logistic function is a kind of maximum entropy distribution, conforming to the decision‐making principle. Under the restriction of synapses, the maximum entropy distribution is the most proper distribution of spike probabilities with the minimum miss‐selection risk. For instance, in the decision‐making process, we make a decision at random without any useful information unless there is sufficient reason to make a choice. Such related inference may answer several issues related to this process.^27^ Learning is a modulation process for decision making. With learning, better choices may be attained in certain situations. To describe this process, Hebb proposed that an increase in synaptic efficacy arises from a presynaptic repeated and persistent stimulation of a postsynaptic cell.^28^ This theory does make sense and is also validated in gastropods.^29^ The similar mechanism for working memory (WM) is short‐term synaptic plasticity (STSP),^30^ and the memory was still considered to be restored in synapses. Taking advantage of PCRAM, we can easily adjust the wiring framework using the cutting‐edge knowledge. For instance, according to Equation (11), there are two main free variables, one is the spike frequency $P_{i}$, and another is the connection matrix *M*. If we want to initially fix spike frequency $P_{i}$ for each neuron to achieve a function, the connection matrix *M* can be solved by Equation (11). Then, we can adjust the PCRAM to change the connection matrix *M* in our device.

How information in WM is maintained is the critical issue to understand the mechanisms underlying WM. Although it is assumed that information in WM is maintained in persistent neuronal activity,^31^, ^32^, ^33^, ^34^, ^35^ some considered the persistent activity is not necessary. For instance, “activity‐silent” memory trace of a stimulus can be maintained in network by STSP.^36^ Besides, recent human studies suggested that information can be encoded in a silent or latent state prior to reactivation into neuronal activity by probing the circuit.^37^, ^38^ In this study, we revealed that the stimulus information can be encoded in artificial neurobionic material in the rest state, just by phase coupling. In addition, the information can also be retrieved by simple modulation. This framework indicates the possibility for WM maintained without persistent activity and even without STSP. Thus, by combination of artificial neurobionic material assemblies, the implementable intelligence devices can achieve more complex functions.

Despite of the findings in this study, the model neurobionic device is still far from clinical application. Essential parts of the device have to include high‐density electrodes for continuous signals input and output, a PCRAM matrix for parameter modulation, and a permanent power system. The major success criteria of this model should be the functional improvements confirmed by neuropsychological tests in the cognitive‐impaired patients, verified by cohort study. Application risks may include the rejection reaction, epileptic effect, and psychiatric disorder. With the progress in PCRAM and biocompatible materials, we will achieve the final success in the near future.

## 5 CONCLUSIONS

In summary, the salient finding from our work proposed a feasible design of an assembly framework based on implementable neurobionic material, reminiscent of real brain tissue. Then, we mathematically validated that the spike probability distribution in this material is a spontaneous phenomenon, and distribution is a maximum entropy distribution under one certain stimulus, which can be applied to decision‐making problem. Second, we also prove that the spike probability fits the solution of a nonlinear equation set, which bridges from connection modification to spike probability. Moreover, we propose the phase coupling effect from parallel to hierarchical neuromorphic assemblies, indicating the possibility of more complex function for these implementable neurobionics devices.
